# Innovative microscale workflow from fungi cultures to Cell Wall‐Degrading Enzyme screening

**DOI:** 10.1111/1751-7915.13405

**Published:** 2019-04-20

**Authors:** Roxane Raulo, Egon Heuson, Ali Siah, Vincent Phalip, Renato Froidevaux

**Affiliations:** ^1^ EA 7394 – ICV – Institut Charles Viollette Univ. Lille 1 ISA INRA Univ. Artois Univ. Littoral Côte d'Opale F‐59000 Lille France

## Abstract

This study aimed at developing a complete miniaturized high‐throughput screening workflow for the evaluation of the Cell Wall‐Degrading Enzyme (CWDE) activities produced by any fungal strain directly cultivated on raw feedstock in a submerged manner. In this study, wheat straw was selected as model substrate as it represents an important carbon source but yet poorly valorised to yield high added value products. Fungi were grown in a microbioreactor in a high‐throughput (HT) way to replace the fastidious shaking flask cultivations. Both approaches were compared in order to validate our new methodology. The range of CWDE activities produced from the cultures was assayed using AZO‐died and pNP‐linked substrates in an SBS plate format using a Biomek FXp pipetting platform. As highlighted in this study, it was shown that the CWDE activities gathered from the microbioreactor cultivations were similar or higher to those obtained from shake flasks cultures, with a lower standard deviation on the measured values, making this new method much faster than the traditional one and suitable for HT CWDE production thanks to its pipetting platform compatibility. Also, the results showed that the enzymatic activities measured were the same when doing the assay manually or using the automated method.

## Introduction

Enzymes secreted by filamentous fungi have a key role in the degradation of the most abundant biopolymers found in nature, that is cellulose and hemicelluloses. These enzymes (Cell Wall‐Degrading Enzymes, CWDE) are of great interest in the industrial conversion of lignocellulosic substrates into component sugars, which then serve as substrates for the synthesis of biofuels and other highly valuable platform molecules. However, the industrial conversion of lignocellulosic biomasses to such monomers faces two major bottlenecks: (i) the plant cell wall is highly recalcitrant to enzymatic degradation due to the presence of lignin which reduces enzyme accessibility to cellulose and hemicelluloses and (ii) a lack of high‐throughput methods to rapidly screen for the enzymatic activities associated to these degradation capabilities in plant‐degrading strains.

Fungi are the predominant source of enzymes currently being used on an industrial scale for the conversion of lignocellulosic biomass to platform molecules (Archer, 2000; Sims *et al*., 2010; Gusakov, 2011). Improving the efficiency of enzymatic saccharification has been an active area of research during the last decade, with efforts dedicated towards the discovery and characterization of novel saccharolytic enzymes (Phalip *et al*., 2005; Carapito *et al*., 2009; Banerjee *et al*., 2010), to be used in biorefineries. A biorefinery is a facility that integrates biomass conversion processes and equipment to produce fuels, power and value‐added chemicals from biomass. As refineries, biorefineries can provide multiples chemicals by fractioning the lignocellulosic biomass into multiple intermediates that can be further converted into value‐added products (Menon and Rao, 2012).The past few years have seen a strong increase in research topics aiming at the isolation and development of novel fungal enzyme mixtures, which can be tailored to efficiently degrade recalcitrant lignocellulose into monomeric sugars with the help of mild or no thermochemical pretreatment (Berrin *et al*., 2012; Couturier *et al*., 2012). Currently, functional screening of fungi is associated with low throughput and prohibitive costs, which severely limits the discovery of novel enzymatic activities and better CWDE producing strains. Fermentation experiments in shake flasks, bench‐scale bioreactors, or a combination of both, are the conventional methods for both fungi screening and fermentation optimization. However, the screening use of these two methods is limited to a small number of strains that can be tested in parallel, as well as the large amount of substrate (feedstock), cultivation medium, sterilization steps, etc. that require such manipulations. Of course, several HT small scale cultivation approaches have already been designed and have proven their efficiency to seek enzyme activities produced by microorganisms. Among them, the recently released microbioreactor device from M2PLabs was extensively used for such application, helping to greatly speed up the cultivation phase. However, when it comes to raw feedstocks as substrates, with filamentous fungi in submerged fermentation, no use of such device has been reported. Developing a screening methodology that would allow for a larger number of cultivations to be performed in parallel directly on raw feedstock, while retaining conditions that can be easily controlled upon scale‐up, would consequently greatly reduce the development time and costs of CWDE cocktails from filamentous fungi, that could later be used to improve the biorefineries’ saccharification processes.

**Figure 1:**
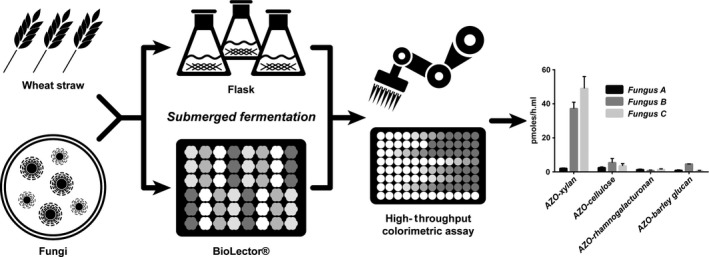
Flow chart of the integrated approach developed for this work with the implementation of high‐throughput (HT) filamentous fungi cultures on wheat straw using the microfermentor microbioreactor followed by HT screening of Cell Wall‐Degrading Enzymes (CWDE) activities using the Biomek FX^P^ (Beckman Coulter, USA) pipetting station. The programming parameters for the BioLector (M2P‐Labs, Germany) and the detailed steps of the Biomek FX^P^ program are described in Appendix S1.

In this study, we present a novel and original approach to screen and select CWDE cocktails from two different fungal strains in a semi‐automated workflow (Fig. 1). The selected strains are *Penicillium chrysogenum*, a saprophytic species that has not been extensively studied for feedstock degradation, and *Zymoseptoria tritici*, the causal agent of wheat blotch. The approach is based on the use of two HT robotic equipment, a Biomek FX^p^ pipetting platform from Beckman Coulter, and the microbioreactor from M2PLabs. This study is the first report of the use of the microbioreactor for the implementation of high‐throughput filamentous fungi cultures on complex biomasses followed by the high‐throughput screening of enzyme activities. This approach is greatly speeding up the screening of fungal CWDE activities by scaling down the whole process to less than 2 ml fermentations. Therefore, with the workflow, we expect to increase the likelihood of discovering interesting strains and enzymes for industrial applications that can directly be used on raw feedstocks in biorefineries.

## Results and discussion

### Automated enzymatic assaying validation

**Figure 2:**
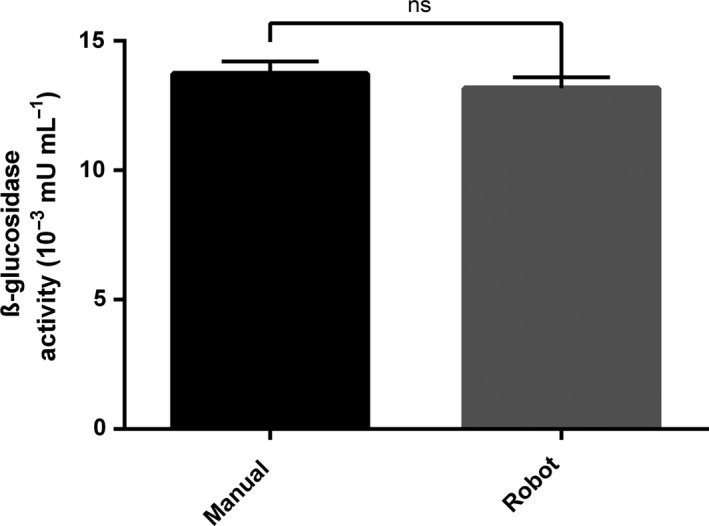
Enzymatic activities measured from the supernatants of *Penicillium chrysogenum* cultures grown on wheat straw for 7 days using the pNP‐glucopyranoside assay. The assays were done manually and using the Biomek FX^P^ pipetting platform. Cultivation supernatants obtained from the microbioreactor were assayed using 4‐Nitrophenyl‐β‐d‐glucopyranoside (pNP‐glucopyranoside, Megazyme) substrate. In a 96 U‐well plate, 30 μl of the culture supernatants were incubated with 150 μl of 0.5% (w/v) pNP substrate solution, 75 μl of 100 mM sodium acetate buffer pH 5.0, completed with water up to a total volume of 300 μl. The reaction mixtures were incubated at room temperature for 2 h with 5 min of initial shaking. The reactions were then stopped by adding 50 μl of the reaction mixture to 200 μl of sodium phosphate solution pH 12 (2% w/v). A total of 100 μl of the supernatants were transferred to a flat‐bottom 96‐well reading plate and the optical densities were measured at 405 nm with a plate reader (SpectraMax i3, Molecular Devices, USA). All experiments were conducted in biological triplicates. Statistical analysis performed did not show any differences between the two conditions.

The automated enzymatic assay results were validated by comparing the relative activity measured when plates were prepared and read manually with the values obtained after the semi‐automated method. It is noteworthy to precise that, for the semi‐automated method, all the pipetting action were performed by the Biomek platform. The only manual step was the centrifugation step. Both methods showed similar results with 13.8 ± 0.48 10^−3^ mUI ml^−1^ and 13.1 ± 0.42 10^−3^ mUI ml^−1^, respectively, measured with the pNP‐glucopyranoside assay when performed manually and with the pipetting station, using wheat straw as substrate and *P. chrysogenum* as fungal strain after 7 days of incubation (Fig. 2).

### Cell Wall‐Degrading Enzyme activities gathered from fungi

**Figure 3:**
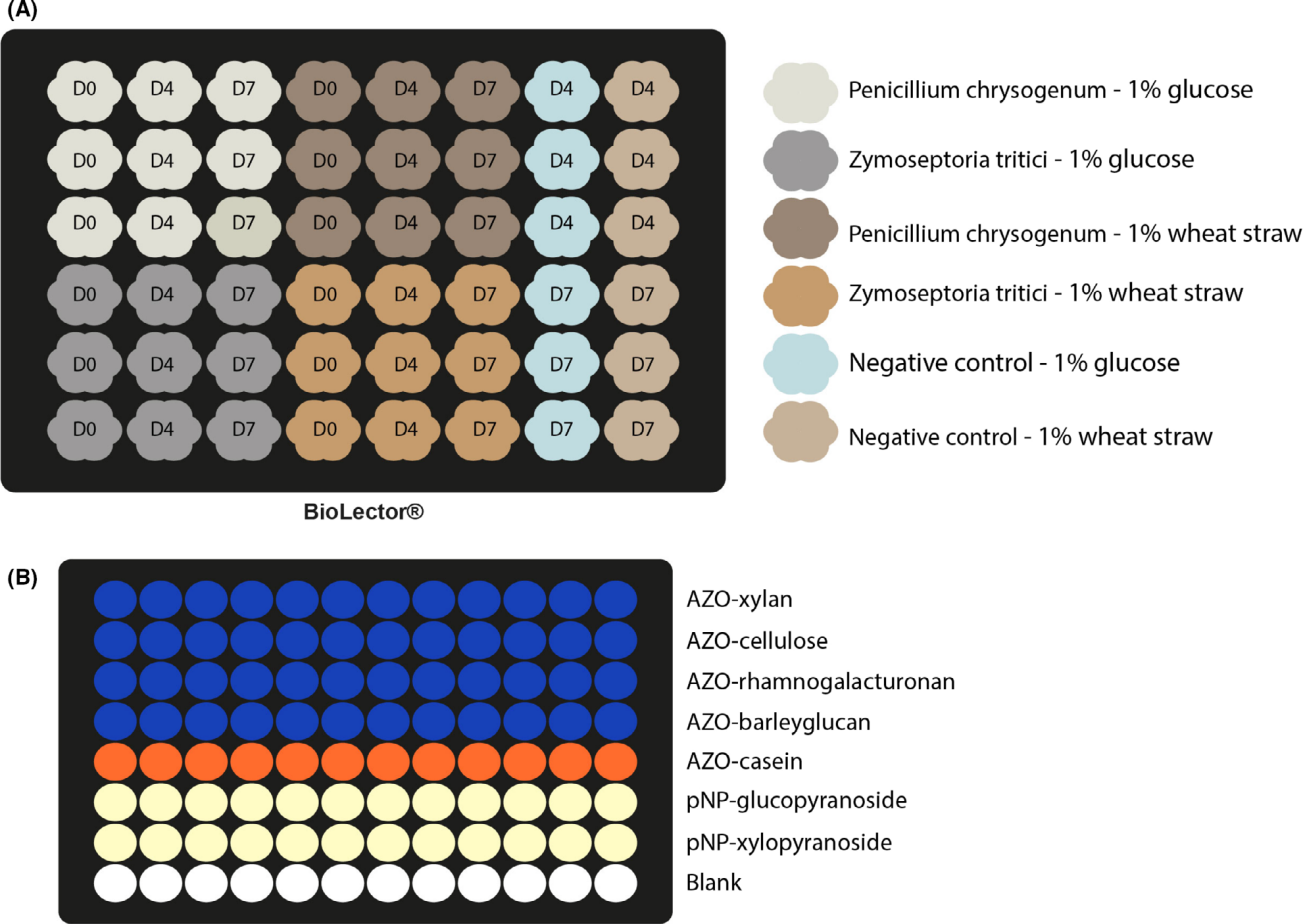
Plates layouts for the microbioreactor cultures as well as the enzymatic assays. A. Cultures in the microbioreactor were done in triplicates (column) for both fungi *Penicilliun chrysogenum* and *Zymoseptoria tritici*. Cultures were carried out for 4 (D4) and 7 days (D7) in minimal medium M3 supplemented with 1% wheat straw. For cultures of *P. chrysogenum* and *Z. tritici* in shake flasks, 5.10^5^ spores per ml in suspension were inoculated into 250‐ml sterile Erlenmeyer flasks containing 100 ml M3 medium (Mitchell *et al*., 1997), supplemented with 1% milled wheat straw (w/v), and incubated for 7 days at 25°C on a shaker (Infors HT Multitron standard, Switzerland) set to 150 r.p.m. Supernatants were manually collected at day 0, day 4 and day 7 after inoculation for enzymatic analysis. For cultures carried out in the BioLector^®^ microbioreactor (M2PLabs, Germany), a FlowerPlate MTP‐48‐B (without optodes) was used as reactor. To lower evaporation and maintain the plate sterility, a low evaporation breathing foil F‐GPR48‐10 was set on top of the plate after filling. Each of the 48 wells was filled with 1.4 ml M3 medium (Mitchell *et al*., 1997) containing 1% milled wheat straw (w/v). The wells were inoculated at 5.10^5^ spores per ml and incubated for 7 days at 25°C at 800 r.p.m. Supernatants were manually collected at day 0, day 4 and day 7 after inoculation for enzymatic analysis by collecting the full content of the well. Feedstock remaining debris and fungi cells were removed from the supernatant through a filtration step using a 0.22 μm syringe filter. The enzymes activities were measured according to the protocol described previously. All experiments were conducted in biological triplicates. Negative control for both substrates was included. B. Enzymatic assays plate layout and results.

**Figure 4:**
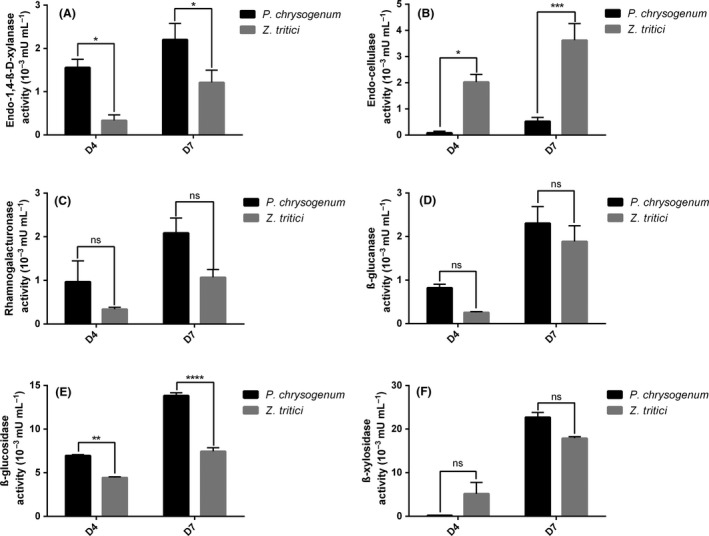
Enzyme activities measured using AZO‐substrates and pNP‐linked substrates after 4 (D4) and 7 days (D7) of cultures in the microbioreactor for *Penicillium chrysogenum* and *Zymoseptoria tritici*. Enzyme activity (A) on AZO‐xylan, (B) on AZO‐cellulose, (C) on AZO‐rhamnogalacturonan, (D) on AZO‐barley glucan, (E) on pNP‐glucopyranoside and (F) on pNP‐xylopyranoside. In a 96 U‐well plate, 30 μl of the culture supernatants were incubated with 150 μl of 0.5% (w/v) of the substrate solution, 75 μl of 100 mM sodium acetate buffer pH 5.0, completed with water up to a total volume of 300 μl. The reaction mixtures were incubated at room temperature for 2 h with 5 min of initial shaking. The reactions were then stopped by adding 50 μl of the reaction mixture to 200 μl of ethanol (95% v/v) (AZO‐xylan, AZO‐cellulose, AZO‐rhamnogalacturonan and AZO‐barley glucan) or sodium phosphate solution pH 12 (2% w/v) (pNP‐glucopyranoside and pNP‐xylopyranoside). The precipitated remaining AZO‐substrates were removed by centrifugation at 2120 *g* for 10 min. A total of 100 μl of the supernatants were transferred to a flat‐bottom 96‐well reading plate and the optical densities were measured at 590 nm and 405 nm, AZO‐substrates and pNP‐substrates, respectively, with a plate reader (SpectraMax i3, Molecular Devices, USA). All experiments were conducted in biological triplicates. A two‐way ANOVA test was performed. * indicates a significant difference within one condition (e.g. D4) when the *P. chrysogenum* and the *Z. tritici* strains were compared (*P* < 0.05).

The capability of the strains used in this study to degrade wheat straw was assayed using a microbioreactor for the implementation of high‐throughput filamentous fungi cultures on complex biomasses followed by the high‐throughput screening of enzyme activities (Fig. 3). Enzymatic activities were assayed after both 4 and 7 days of cultures on wheat straw of *P. chrysogenum* and *Z. tritici*. For all the enzymatic assays tested, an increase in enzymatic activities was observed between the D4 and D7 time points (Fig. 4). Using this semi‐automated method, the tests assayed (Table 1) were able to discriminate for specific activities for each fungus. The AZO‐cellulose test showed higher activities measurements for *Z. tritici* while the other tests showed that *P. chrysogenum* was more efficient at degrading these substrates. In addition, the method allowed the measurement of low enzyme activities, the lowest being 0.18 10^−3^ mUI ml^−1^ for *P. chrysogenum* on the pNP‐xylopyranoside substrate. These results, and the sensitivity of the assays, demonstrated the potential for the developed method to be used for HT screening of CWDEs on complex substrates.

**Table 1:** List of the enzymatic assays used in this study

Test name	Activity tested	Reference manufacturer
AZO‐xylan (Birchwood)	*endo*‐1,4‐ß‐d‐xylanase	Megazyme S‐AXBP
AZO‐cellulase	*endo*‐1,4‐ß‐d‐glucanase	Megazyme S‐ACMC
AZO‐rhamnogalacturonan	Rhamnogalacturonan lyase	Megazyme S‐AZRH
AZO‐barley glucan	β‐glucanase	Megazyme S‐ABG100
pNP‐glucopyranoside	*exo*‐1,4‐ß‐d‐glucanase	Megazyme O‐PNPBG
pNP‐xylospyranoside	*exo*‐1,4‐ß‐d‐xylanase	Megazyme O‐PNPX

### Performance comparison between the microbioreactor and the flask cultures

**Figure 5:**
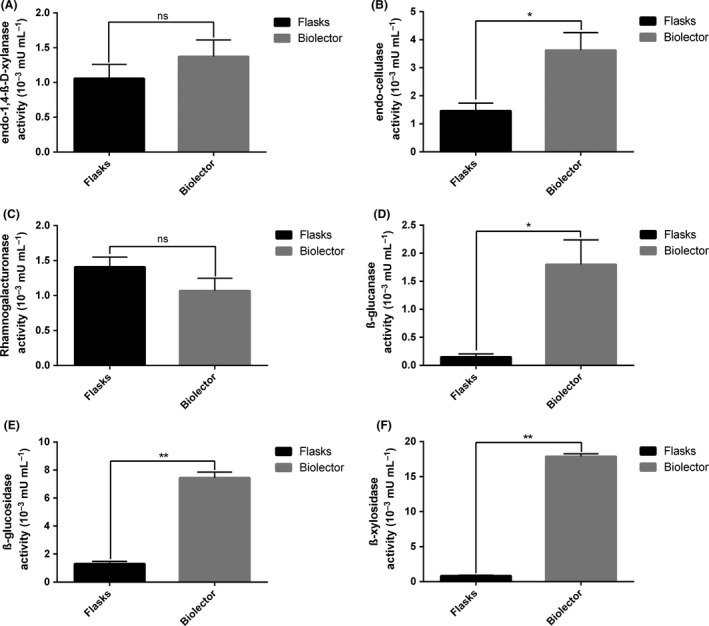
Enzyme activities measured using AZO‐substrates and pNP‐linked substrates after 7 days of cultures in shake flask cultures and in the microbioreactor for *Zymoseptoria tritici*. Enzyme activity (A) on AZO‐xylan, (B) on AZO‐cellulose, (C) on AZO‐rhamnogalacturonan, (D) on AZO‐barley glucan, (E) on pNP‐glucopyranoside and (F) on pNP‐xylopyranoside. For cultures of *P. chrysogenum* and *Z. tritici* in shake flasks, spores were washed with 0.01% (v/v) Tween‐80 solution and 5.10^5^ spores per ml in suspension were inoculated into 250 ml sterile Erlenmeyer flasks containing 100 ml M3 medium (Mitchell *et al*., 1997), supplemented with 1% milled wheat straw (w/v), and incubated for 7 days at 25°C on a shaker (Infors HT Multitron standard, Switzerland) set to 150 r.p.m. Media were autoclaved at 110°C for 30 min instead of 121°C to limit the Maillard reaction. Supernatants were manually collected at day 0, day 4 and day 7 after inoculation for enzymatic analysis. Feedstock remaining debris and fungi cells were removed from the supernatant through a filtration step using a 0.22 μm syringe filter. All experiments were conducted in biological triplicates. The enzymes activities were measured according to the protocol described previously. A *t*‐test was performed for statistical analysis. * indicates a significant difference between the activity measured in the shake flask and in the microbioreactor (*P* < 0.05).

The comparison in enzymatic activities measured in shake flask cultures compared to the one obtained using the microbioreactor was performed with the *Z. tritici* strain over a time course of 7 days. When comparing the data obtained from both the cultures in shake flasks and in the microbioreactor, the trend in CWDE activities measured was the same with the 5 AZO‐substrates, all along the culture. For instance, at day 7, when considering the AZO‐xylan substrate, the same levels of enzyme activities were measured for two types of culture, with 1.06 10^−3^ mUI ml^−1^ and 1.37 10^−3^ mUI ml^−1^ measured in the shake flask cultures and in the microbioreactor respectively (Fig. 5). The most significant results were obtained for the pNP‐linked substrates, with an increase in the enzyme activities measured for the two types of pNP‐substrates in the microbioreactor which may be due to a better oxygen transfer rate in the microbioreactor (Fig. 5). These results highlight the potential of the described method for HT screening as the cultures volume was reduced by a factor 70 while allowing for differential enzyme activities measurement. In addition, HT screening of the enzyme activities was also achieved with the use of the Beckman Coulter robot.

## Conclusion

When designing an industrial fermentation, a large number of experiments are required to respectively select a strain, improve the cultivation medium and optimize the fermentation procedure, measure the enzymes activities and finally select the most efficient strain for the degradation of a specific biomass. The limitations of shake flasks cultures like the inability to control parameters, other than agitation rate and temperature, usually means that the scale‐up to the bioreactor scale is rather complicated as it is neglecting, for instance, the effect of oxygen supply on growth and the metabolite production during fermentation. For the first time, these results highlighted the potential of the microbioreactor to screen filamentous fungi species on raw feedstocks since similar and higher CWDE activities were observed from the microbioreactor cultures compared to the shake flask ones. These cultivations were done directly using shredded wheat straw as raw substrate in a submerged manner, proving the ability of the equipment to run accurate cultivations on such complex material with some advantages compared to the current flask cultures method (i.e. more conditions screened, less amount of substrate needed, better activities as shown in Fig. 4). In addition, the scale‐up from small scale to large scale bioreactor is often more predictable than from shake flasks to bioreactor (Rohe *et al*., 2012). Overall, the differences in activities measured in this work were strong enough to discriminate between the screened strains with different responses obtained depending on the substrate assayed. Using this tool, an increased number of strains and parameters will be tested in a close future, setting this workflow as an efficient starting point for the discovery of fungal CWDE cocktails that could later be scaled up to an industrial production level for biorefineries purpose. This method is currently being upgraded as a fully automated process with the sampling of the supernatants done directly in the wells using a Beckman Coulter robot (Biomek NX^p^ with a BioLector Pro^®^ integrated to the deck).

## Conflict of interest

None declared.

## Supporting information


**Appendix S1.** Fungal strains cultivation in the BioLector.
