# Supplementary material for: Innovative microscale workflow from fungi cultures to Cell Wall‐Degrading Enzyme screening
Source: Microb Biotechnol. 2019 Apr 20;12(6):1286–92. doi: 10.1111/1751-7915.13405 (PMC6801129; doi:10.1111/1751-7915.13405)
Supplement: Supplementary file 1 — Appendix S1. Fungal strains cultivation in the BioLector. [file MBT2-12-1286-s001.docx]

- **Fungal strains cultivation in the BioLector**

For fungi cultivation, the BioLector I was set up using the following parameters. A 48-wells FlowerPlate with pH optode (MTP-48-BH) was selected in step 2 (Fig. 1). The lot number was entered according to the number written on the plate package. The plate layout was defined as 48 different samples in step 3 (Fig. 2). The incubation parameters were defined in step 4 (Fig. 3), with 800 rotation per minute as agitation, and a 25 °C incubation temperature. The humidity control was also set to on during this step. Gas control was not activated. In step 5 (Fig. 4), 6 filters were applied. The five first filters were set to biomass measurement using the light scattering system with different gains (10, 25, 50, 75 and 100 respectively, according to the manufacturer instructions). The last filter was set on pH measurement mode. In the final step (Fig. 5), the duration between to measurement of the previous parameters was set to 15 min, and the experiment end time to manual.


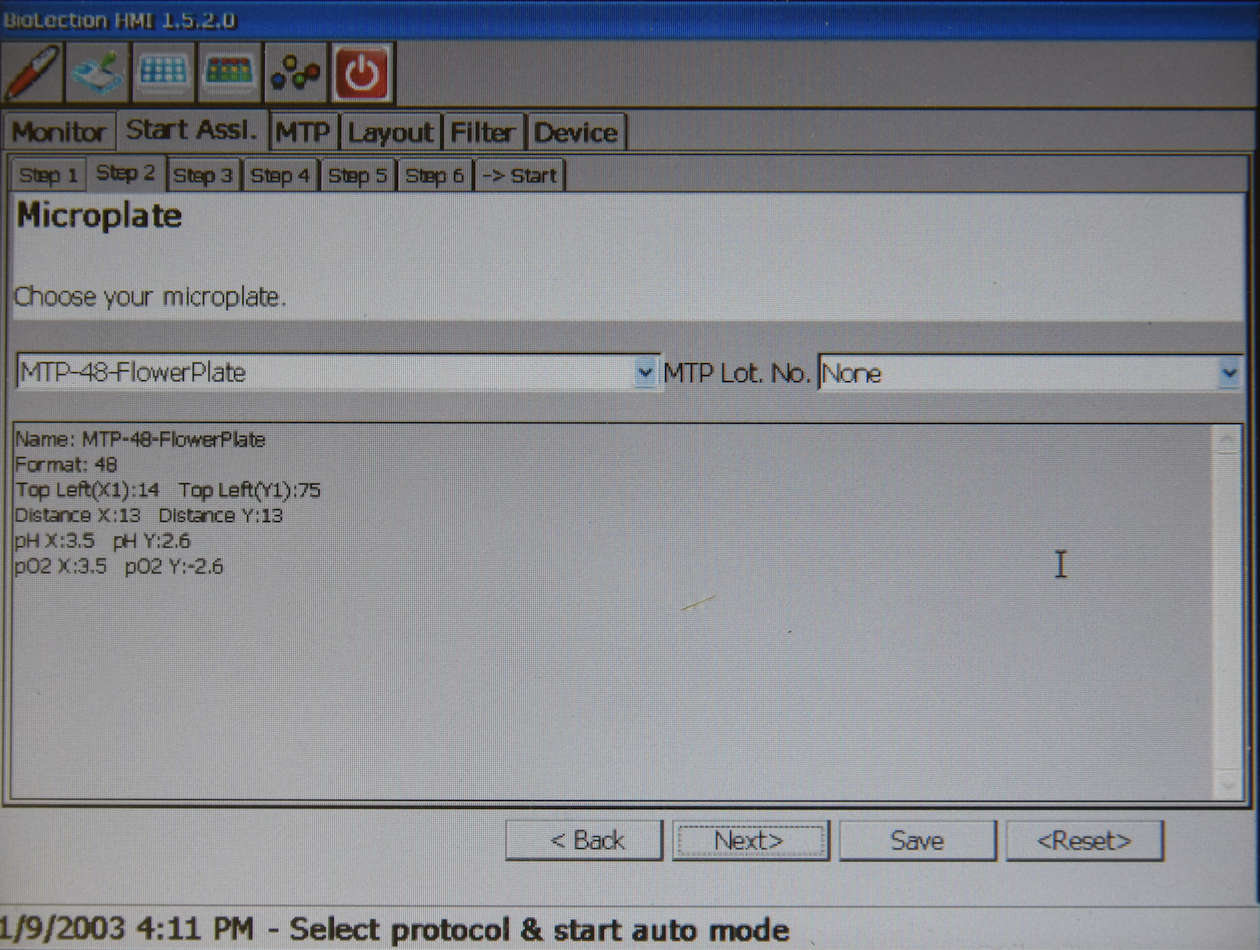


Fig. 1: 2^sd^ step of BioLector programming. The first step corresponds to the program’s name selection.


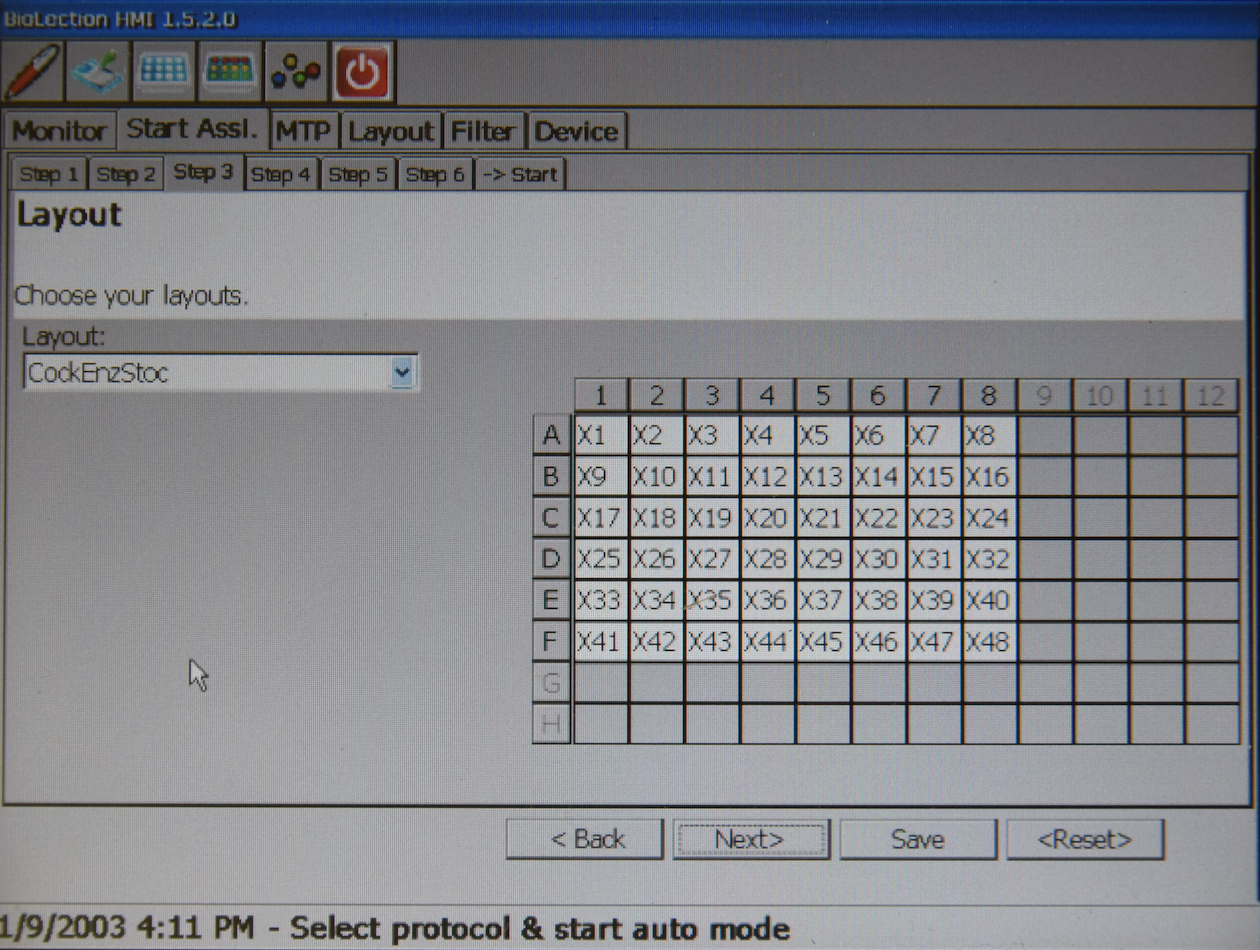


Fig. 2: 3^rd^ step of BioLector programming.


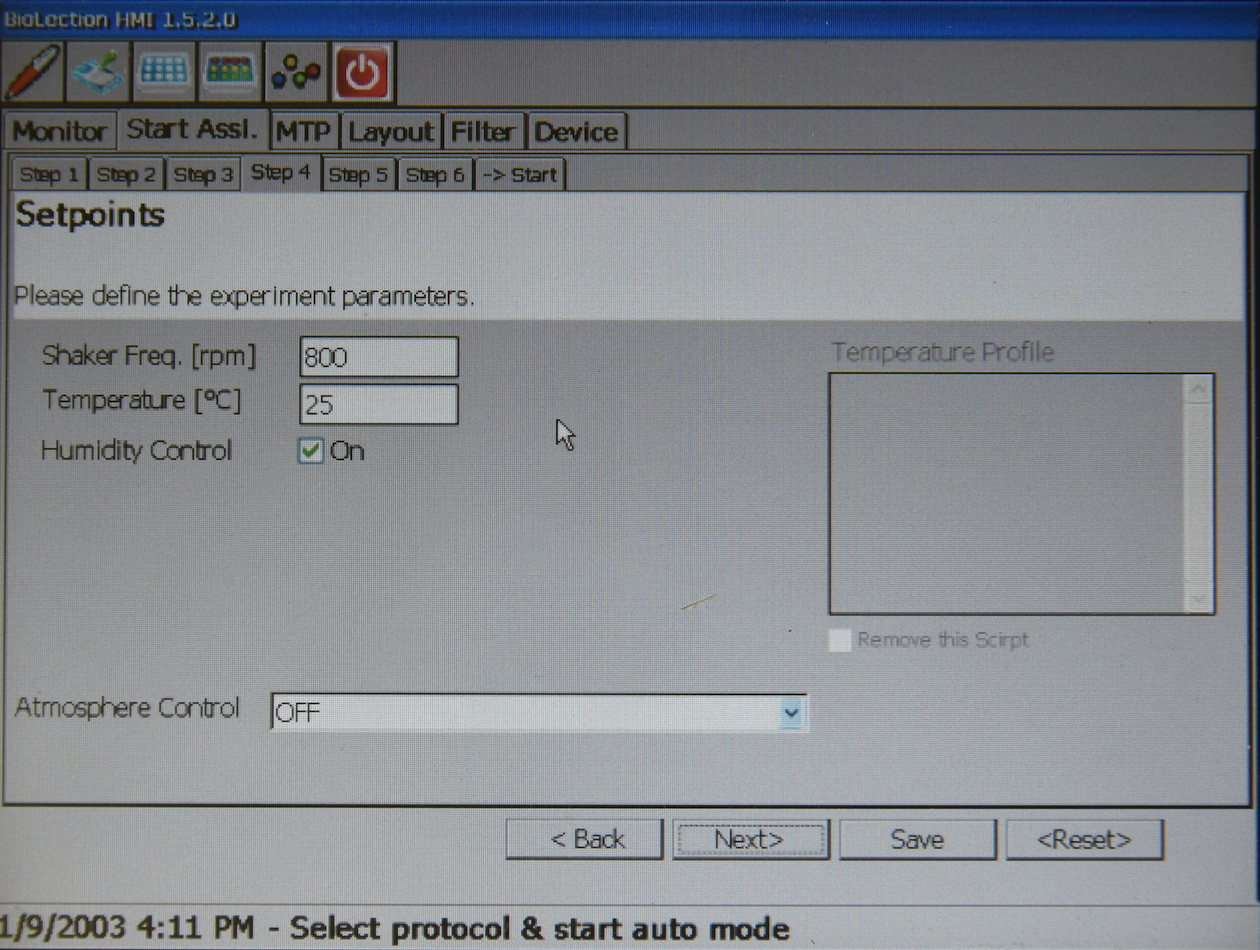


Fig. 3: 4^th^ step of BioLector programming


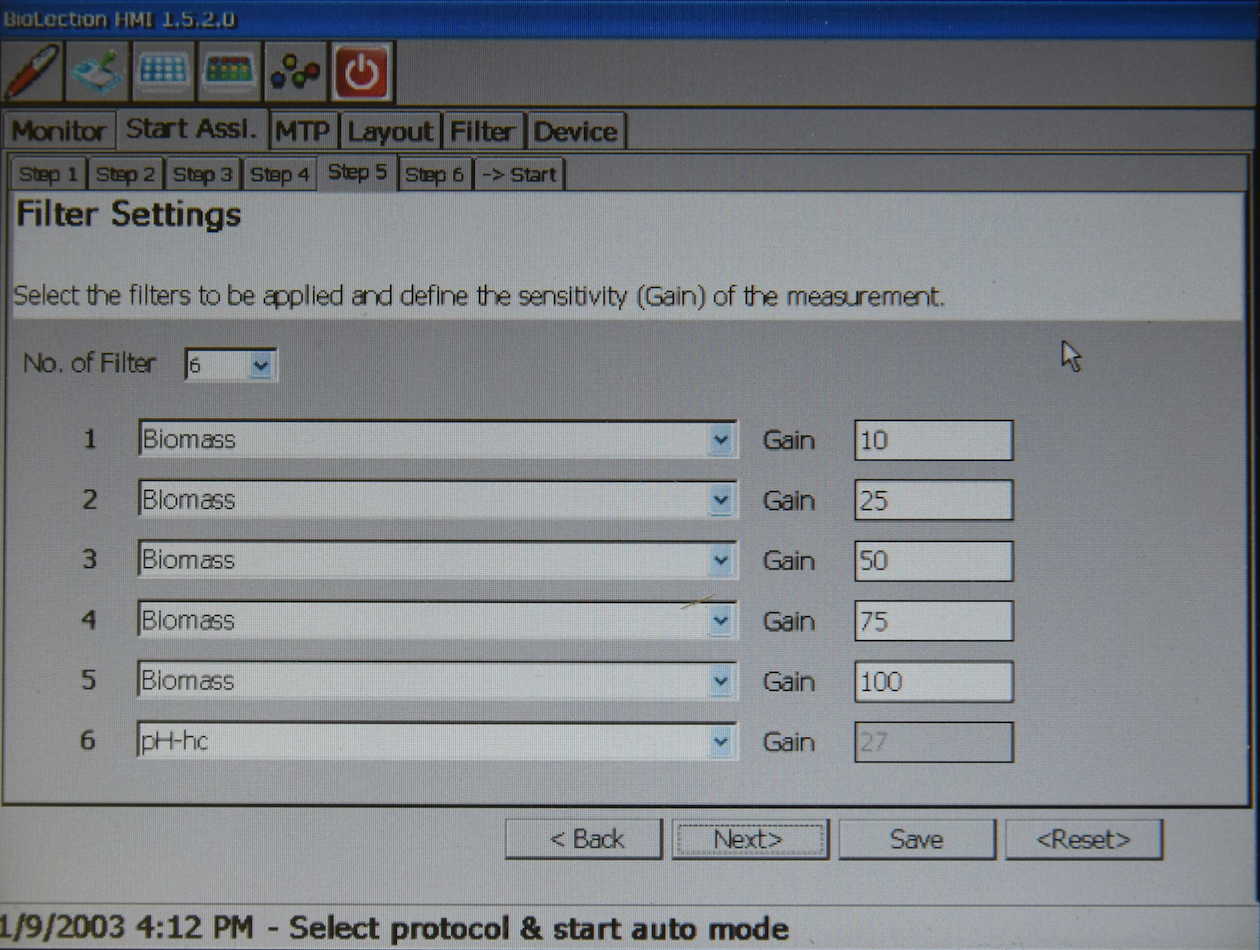


Fig. 4: 5^th^ step of BioLector programming


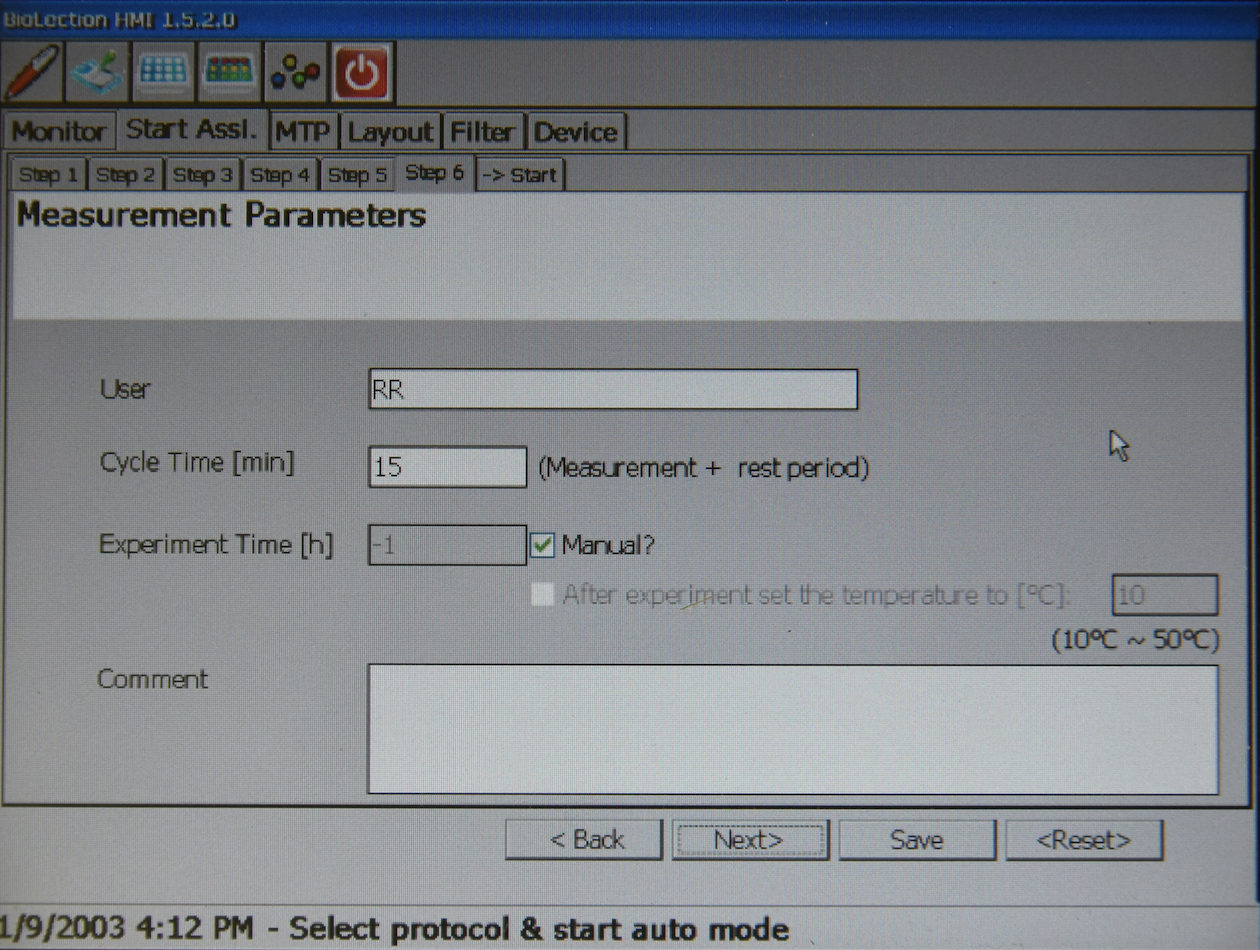


Fig. 5: 6^th^ step of BioLector programming

- **Beckman Coulter FX^p^ program for substrate plate preparation**

---------------------------------------------------------------------------

**Start**

---------------------------------------------------------------------------

Comment

Description:

**Initial Setup**

Comment:

Plates:

"**Substrats**": ---

Reservoirs:

"**2*Substrats+1*H2O+1*TpAc**":

MicroTube1: AZO-xylan - 2000uL

MicroTube7: AZO-cellulose - 2000uL

MicroTube13: AZO-barleyglucan - 2000uL

MicroTube19: AZO-rhamnogalacturonan - 2000uL

MicroTube2: AZO-casein - 2000uL

MicroTube8: pNP-glucopyranoside - 2000uL

MicroTube14: pNP-xylopyranoside - 2000uL

MicroTube20: H2O - 2000uL

MicroTube3: H2O - 2000uL

MicroTube9: H2O - 2000uL

MicroTube15: H2O - 2000uL

MicroTube21: H2O - 2000uL

MicroTube4: Acetate buffer pH5 - 2000uL

MicroTube10: Acetate buffer pH5 - 2000uL

MicroTube16: Acetate buffer pH5 - 2000uL

MicroTube22: Acetate buffer pH5 - 2000uL

"**12*Enzymes**"

MicroTube1: Supernatant1 R1 - 1000uL

MicroTube2: Supernatant1 R2 - 1000uL

MicroTube3: Supernatant1 R3 - 1000uL

MicroTube4: Supernatant2 R1 - 1000uL

MicroTube5: Supernatant2 R2 - 1000uL

MicroTube6: Supernatant2 R3 - 1000uL

MicroTube7: Supernatant3 R1 - 1000uL

MicroTube8: Supernatant3 R2 - 1000uL

MicroTube9: Supernatant3 R3 - 1000uL

MicroTube10: Supernatant4 R1 - 1000uL

MicroTube11: Supernatant4 R2 - 1000uL

MicroTube12: Supernatant4 R3 - 1000uL

---------------------------------------------------------------------------

**Instrument Setup**

Deck: REALCAT_Biomek_HTS

Pause to confirm layout.

Verify that the pod is set up in its default configuration.

Items:

AW1: Water

FBBCR1: Nothing

MultimodeReader1: Nothing

P1: Nothing

P10: Nothing

P11: Nothing

P12: Nothing

P13: Nothing

P14: Nothing

P2: Nothing

P3: Nothing

P4: Portoir_Eppendorf_2000uL named 2*Substrats+1*H2O+1*TpAc with known volumes: 0 µL of Water in A5..A6, A11..A12, A17..A18, A23..A24, 2000 µL of Water in A1..A4, A7..A10, A13..A16, A19..A22

P5: Plate_96_U_PS_Greiner named Substrats with known volume: 0 µL of Water in all wellslid_standard_greiner named Lid_Substrats

P6: Nothing

P7: Nothing

P8: Nothing

P9: Portoir_Eppendorf_1500uL named 12*Enzymes with known volumes: 0 µL of Water in

A13..A24, 1000 µL of Water in A1..A12

PP1: Nothing

PeltFlat_1: Nothing

SPeltFlat_1: Nothing

TL1: Nothing

TR1: Nothing

inner1: Nothing

outer1: Nothing

---------------------------------------------------------------------------

**Span-8 Wash**

Wash all probes of Pod2 at AW1; do a passive wash by dispensing 8 mL while in the wells

and 2 mL to waste. Delay for 300 ms after each dispense.

---------------------------------------------------------------------------

**Move Labware**

Move the top "1" plates at "P5" to "P3" using pod "Pod1".

---------------------------------------------------------------------------

**Group:Water**

---------------------------------------------------------------------------

- **Loop**

Loop from "b" = "1" to "12", incrementing by "1".

---------------------------------------------------------------------------

Span-8 Aspirate

Using Pod2, Aspirate 45 µL from the labware at 2*Substrats+1*H2O+1*TpAc using an auto-selected technique.

Probes 1-4 will be used, with a spacing of 1.

The first selected probe will pipette from well 3.

Override the technique height by moving to -5 mm from the liquid.

The liquid type is Water and the expected labware type is Portoir_Eppendorf_2000uL.

---------------------------------------------------------------------------

Span-8 Aspirate

Using Pod2, Aspirate 45 µL from the labware at 2*Substrats+1*H2O+1*TpAc using an auto-selected technique.

Probes 5-8 will be used, with a spacing of 1.

The first selected probe will pipette from well 3.

Override the technique height by moving to -5 mm from the liquid.

The liquid type is Water and the expected labware type is Portoir_Eppendorf_2000uL.

---------------------------------------------------------------------------

Span-8 Dispense

Using Pod2, Dispense 45 µL to the labware at Substrats using an auto-selected technique.

All probes will be used, with a spacing of 1.

The first selected probe will pipette to well = b.

Override the technique height by moving to -1 mm from the liquid.

The liquid type is Water and the expected labware type is Plate_96_U_PS_Greiner.

---------------------------------------------------------------------------

- **End Loop**

---------------------------------------------------------------------------

Span-8 Wash

Wash all probes of Pod2 at AW1; do a passive wash by dispensing 5 mL while in the wells

and 2 mL to waste. Delay for 300 ms after each dispense.

---------------------------------------------------------------------------

**End Group**

---------------------------------------------------------------------------

**Group:Buffer**

---------------------------------------------------------------------------

- **Loop**

Loop from "c" = "1" to "12", incrementing by "1".

---------------------------------------------------------------------------

Span-8 Aspirate

Using Pod2, Aspirate 75 µL from the labware at 2*Substrats+1*H2O+1*TpAc using an auto-selected technique.

Probes 1-4 will be used, with a spacing of 1.

The first selected probe will pipette from well 4.

Override the technique height by moving to -5 mm from the liquid.

Projects\CockEnzStoc\20171018_CockEnzStoc_Order1_Substrate-Enzyme

12/7/2017 2:49:55 PM Page:3

The liquid type is Water and the expected labware type is Portoir_Eppendorf_2000uL.

---------------------------------------------------------------------------

Span-8 Aspirate

Using Pod2, Aspirate 75 µL from the labware at 2*Substrats+1*H2O+1*TpAc using an auto-selected technique.

Probes 5-8 will be used, with a spacing of 1.

The first selected probe will pipette from well 4.

Override the technique height by moving to -5 mm from the liquid.

The liquid type is Water and the expected labware type is Portoir_Eppendorf_2000uL.

---------------------------------------------------------------------------

Span-8 Dispense

Using Pod2, Dispense 75 µL to the labware at Substrats using an auto-selected technique.

All probes will be used, with a spacing of 1.

The first selected probe will pipette to well = c.

Override the technique height by moving to 0.1 mm from the liquid.

The liquid type is Water and the expected labware type is Plate_96_U_PS_Greiner.

---------------------------------------------------------------------------

Span-8 Wash

Wash all probes of Pod2 at AW1; do a passive wash by dispensing 2 mL while in the wells

and 2 mL to waste. Delay for 300 ms after each dispense.

---------------------------------------------------------------------------

- **End Loop**

---------------------------------------------------------------------------

Span-8 Wash

Wash all probes of Pod2 at AW1; do a passive wash by dispensing 5 mL while in the wells

and 2 mL to waste. Delay for 300 ms after each dispense.

---------------------------------------------------------------------------

**End Group**

---------------------------------------------------------------------------

**Group:Substrates**

---------------------------------------------------------------------------

- **Loop**

Loop from "a" = "1" to "12", incrementing by "1".

---------------------------------------------------------------------------

Span-8 Aspirate

Using Pod2, Aspirate 150 µL from the labware at 2*Substrats+1*H2O+1*TpAc using an auto-selected technique.

Probes 1-4 will be used, with a spacing of 1.

The first selected probe will pipette from well 1.

Override the technique height by moving to -5 mm from the liquid.

The liquid type is Water and the expected labware type is Portoir_Eppendorf_2000uL.

---------------------------------------------------------------------------

Span-8 Aspirate

Using Pod2, Aspirate 150 µL from the labware at 2*Substrats+1*H2O+1*TpAc using an auto-selected technique.

Probes 5-8 will be used, with a spacing of 1.

The first selected probe will pipette from well 2.

Override the technique height by moving to -5 mm from the liquid.

The liquid type is Water and the expected labware type is Portoir_Eppendorf_2000uL.

---------------------------------------------------------------------------

Span-8 Dispense

Using Pod2, Dispense 150 µL to the labware at Substrats using an auto-selected technique.

All probes will be used, with a spacing of 1.

The first selected probe will pipette to well = a.

Override the technique height by moving to 0.1 mm from the liquid.

The liquid type is Water and the expected labware type is Plate_96_U_PS_Greiner.

---------------------------------------------------------------------------

Span-8 Wash

Wash all probes of Pod2 at AW1; do a passive wash by dispensing 2 mL while in the wells

and 2 mL to waste. Delay for 300 ms after each dispense.

---------------------------------------------------------------------------

- **End Loop**

---------------------------------------------------------------------------

Span-8 Wash

Wash all probes of Pod2 at AW1; do a passive wash by dispensing 5 mL while in the wells

and 5 mL to waste. Delay for 300 ms after each dispense.

---------------------------------------------------------------------------

**End Group**

---------------------------------------------------------------------------

**Group:Enzyme**

---------------------------------------------------------------------------

- **Loop**

Loop from "d" = "1" to "12", incrementing by "1".

---------------------------------------------------------------------------

- **Loop**

Loop from "e" = "1" to "8", incrementing by "1".

---------------------------------------------------------------------------

Span-8 Aspirate

Using Pod2, Aspirate 30 µL from the labware at 12*Enzymes using an auto-selected technique.

Probes specified by "= e" will be used, with a spacing of 1.

The first selected probe will pipette from well = d.

Override the technique height by moving to -5 mm from the liquid.

The liquid type is Water and the expected labware type is Portoir_Eppendorf_1500uL.

---------------------------------------------------------------------------

- **End Loop**

---------------------------------------------------------------------------

Span-8 Dispense

Using Pod2, Dispense 30 µL to the labware at Substrats using the following technique:

Use the following pipetting template: Span-8

Calibration Offset: 0.353

Calibration Slope: 1.08

Minimum Pipetting Height: 0.5 mm

Prewet: False

Blowout: True

Follow Liquid: True

Height: 1.5 mm from the liquid

Mix: True

Mix Aspirate Speed: 100µL/s

Mix Aspirate Height: 1.5 mm from the bottom

Mix Dispense Speed: 400µL/s

Mix Dispense Height: -1.5 mm from the liquid

Mix Count: 5

Mix Volume: 100 µL

Operation speed: 100µL/s

Tip Touch: False

All probes will be used, with a spacing of 1.

The first selected probe will pipette to well = d.

Override the technique height by moving to -1.5 mm from the liquid.

The liquid type is Water and the expected labware type is Plate_96_U_PS_Greiner.

---------------------------------------------------------------------------

Span-8 Wash

Wash all probes of Pod2 at AW1; do a passive wash by dispensing 4 mL while in the wells and 2 mL to waste. Delay for 300 ms after each dispense.

---------------------------------------------------------------------------

- **End Loop**

---------------------------------------------------------------------------

**End Group**

---------------------------------------------------------------------------

**Move Labware**

Move the entire stack of labware at "P3" to "P5" using pod "Pod1".

---------------------------------------------------------------------------

**Span-8 Wash**

Wash all probes of Pod2 at AW1; do a passive wash by dispensing 8 mL while in the wells

and 2 mL to waste. Delay for 300 ms after each dispense.

---------------------------------------------------------------------------

**Finish**

Method completed.

Remove the tips from all pods. Clear all labware from the deck. Clear all labware from SILAS devices. Clear all global variables.

- **Beckman Coulter FX^p^ program for precipitation plate preparation**

---------------------------------------------------------------------------

**Start**

---------------------------------------------------------------------------

SpectraMaxI3: Initialize the device, readying it for automated use.

---------------------------------------------------------------------------

Comment

Description:

**Initial Setup**

Comment:

Plates:

"**Substrats**": ---

Reservoirs:

"**4*Neutralisant**":

MicroTube1: BSA 0.1mg/mL - 1000uL

MicroTube7: BSA 0.2mg/mL - 1000uL

MicroTube13: BSA 0.3mg/mL - 1000uL

MicroTube19: BSA 0.4mg/mL - 1000uL

MicroTube2: BSA 0.5mg/mL - 1000uL

MicroTube8: BSA 0.6mg/mL - 1000uL

MicroTube14: BSA 0.7mg/mL - 1000uL

MicroTube20: BSA 0.8mg/mL - 1000uL

MicroTube3: BSA 0.9mg/mL - 1000uL

MicroTube9: BSA 1.0mg/mL - 1000uL

MicroTube15: BSA 0mg/mL - 1000uL

MicroTube21: BSA 0mg/mL - 1000uL

MicroTube4: BSA 0mg/mL - 1000uL

MicroTube10: BSA 0mg/mL - 1000uL

MicroTube16: BSA 0mg/mL - 1000uL

MicroTube22: BSA 0mg/mL - 1000uL

---------------------------------------------------------------------------

Instrument Setup

Deck: REALCAT_Biomek_HTS

Pause to confirm layout.

Verify that the pod is set up in its default configuration.

Items:

AW1: Water

FBBCR1: Nothing

MultimodeReader1: Nothing

P1: Nothing

P10: Nothing

P11: Nothing

P12: Nothing

P13: Nothing

P14: Nothing

P2: Nothing

P3: Plate_96_U_PS_Greiner named Samples with an unknown volume of Water in all

wells.lid_standard_greiner named Lid_Samples

P4: Portoir_Eppendorf_1500uL named 4*Neutralisant with known volumes: 1400 µL of Organic in A1, A3, A7, A9, A13, A15, 0 µL of Water in A5..A6, A11..A12, A17..A18, A23..A24, 1400µL of Water in A2, A4, A8, A10, A14, A16, A19..A22

P5: Plate_96_U_PS_Greiner named Neutralisation with known volume: 0 µL of Water in all wellslid_standard_greiner named Lid_Neutralisation

P6: Nothing

P7: Nothing

P8: Nothing

P9: Plate_96_F_Vis_Greiner named Lecture with known volume: 0 µL of Water in all wellslid_standard_greiner named Lid_Lecture

PP1: Nothing

PeltFlat_1: Nothing

SPeltFlat_1: Nothing

TL1: Nothing

TR1: Nothing

inner1: Nothing

outer1: Nothing

---------------------------------------------------------------------------

**Span-8 Wash**

Wash all probes of Pod2 at AW1; do a passive wash by dispensing 8 mL while in the wells

and 2 mL to waste. Delay for 300 ms after each dispense.

---------------------------------------------------------------------------

**Move Labware**

Move the top "1" plates at "P5" to "P6" using pod "Pod1".

---------------------------------------------------------------------------

**Group: Neutralisants**

---------------------------------------------------------------------------

- **Loop**

Loop from "b" = "0" to "1", incrementing by "1".

---------------------------------------------------------------------------

- **Loop**

Loop from "a" = "1" to "6", incrementing by "1".

---------------------------------------------------------------------------

Span-8 Aspirate

Using Pod2, Aspirate 200 µL from the labware at 4*Neutralisant using an auto-selected technique.

Probes 1-4 will be used, with a spacing of 1.

The first selected probe will pipette from well = 1 + b * 2.

Override the technique height by moving to -5 mm from the liquid.

The liquid type is Organic and the expected labware type is Portoir_Eppendorf_1500uL.

---------------------------------------------------------------------------

Span-8 Aspirate

Using Pod2, Aspirate 200 µL from the labware at 4*Neutralisant using an auto-selected technique.

Probes 5-8 will be used, with a spacing of 1.

The first selected probe will pipette from well = 2 + b * 2.

Override the technique height by moving to -5 mm from the liquid.

The liquid type is Organic and the expected labware type is Portoir_Eppendorf_1500uL.

---------------------------------------------------------------------------

Span-8 Dispense

Using Pod2, Dispense 200 µL to the labware at Neutralisation using an auto-selected technique.

All probes will be used, with a spacing of 1.

The first selected probe will pipette to well = a + b * 6.

Override the technique height by moving to 0.1 mm from the liquid.

The liquid type is Organic and the expected labware type is Plate_96_U_PS_Greiner.

---------------------------------------------------------------------------

- **End Loop**

---------------------------------------------------------------------------

- **End Loop**

---------------------------------------------------------------------------

Span-8 Wash

Wash all probes of Pod2 at AW1; do a passive wash by dispensing 5 mL while in the wells and 5 mL to waste. Delay for 300 ms after each dispense.

---------------------------------------------------------------------------

**End Group**

---------------------------------------------------------------------------

**Move Labware**

Move the top "1" plates at "P3" to "P2" using pod "Pod1".

---------------------------------------------------------------------------

**Group: Neutralisation**

---------------------------------------------------------------------------

- **Loop**

Loop from "c" = "1" to "12", incrementing by "1".

---------------------------------------------------------------------------

Span-8 Aspirate

Using Pod2, Aspirate 50 µL from the labware at Samples using an auto-selected technique.

All probes will be used, with a spacing of 1.

The first selected probe will pipette from well = c.

Override the technique height by moving to 2 mm from the bottom.

The liquid type is Water and the expected labware type is Plate_96_U_PS_Greiner.

Projects\CockEnzStoc\20171018_CockEnzStoc_Order2_Neutralisation-Lecture

12/7/2017 2:53:01 PM Page:3

---------------------------------------------------------------------------

Span-8 Dispense

Using Pod2, Dispense 50 µL to the labware at Neutralisation using the following technique:

Use the following pipetting template: Span-8

Calibration Offset: 0.353

Calibration Slope: 1.08

Minimum Pipetting Height: 0.5 mm

Prewet: False

Blowout: True

Follow Liquid: True

Height: 1.5 mm from the liquid

Mix: True

Mix Aspirate Speed: 100µL/s

Mix Aspirate Height: 1.5 mm from the bottom

Mix Dispense Speed: 400µL/s

Mix Dispense Height: -1.5 mm from the liquid

Mix Count: 3

Mix Volume: 50 µL

Operation speed: 100µL/s

Tip Touch: False

All probes will be used, with a spacing of 1.

The first selected probe will pipette to well = c.

Override the technique height by moving to -1.5 mm from the liquid.

The liquid type is Water and the expected labware type is Plate_96_U_PS_Greiner.

---------------------------------------------------------------------------

Span-8 Wash

Wash all probes of Pod2 at AW1; do a passive wash by dispensing 4 mL while in the wells and 2 mL to waste. Delay for 300 ms after each dispense.

---------------------------------------------------------------------------

- **End Loop**

---------------------------------------------------------------------------

**End Group**

---------------------------------------------------------------------------

**Move Labware**

Move the entire stack of labware at "P2" to "P3" using pod "Pod1".

---------------------------------------------------------------------------

**Move Labware**

Move the entire stack of labware at "P6" to "P5" using pod "Pod1".

---------------------------------------------------------------------------

**Span-8 Wash**

Wash all probes of Pod2 at AW1; do a passive wash by dispensing 10 mL while in the wells and 10 mL to waste. Delay for 300 ms after each dispense.

---------------------------------------------------------------------------

**Pause**

Pause everything while waiting for a user response to the following prompt: "Paused".

---------------------------------------------------------------------------

**Move Labware**

Move the top "1" plates at "P5" to "P6" using pod "Pod1".

---------------------------------------------------------------------------

**Move Labware**

Move the top "1" plates at "P9" to "P10" using pod "Pod1".

---------------------------------------------------------------------------

**Group: Transfert**

---------------------------------------------------------------------------

- **Loop**

Loop from "d" = "1" to "12", incrementing by "1".

---------------------------------------------------------------------------

Span-8 Aspirate

Using Pod2, Aspirate 100 µL from the labware at Neutralisation using an auto-selected technique.

All probes will be used, with a spacing of 1.

The first selected probe will pipette from well = d.

Override the technique height by moving to -2 mm from the liquid.

The liquid type is Organic and the expected labware type is Plate_96_U_PS_Greiner.

---------------------------------------------------------------------------

Span-8 Dispense

Projects\CockEnzStoc\20171018_CockEnzStoc_Order2_Neutralisation-Lecture

12/7/2017 2:53:01 PM Page:4

Using Pod2, Dispense 100 µL to the labware at Lecture using an auto-selected technique.

All probes will be used, with a spacing of 1.

The first selected probe will pipette to well = d.

Override the technique height by moving to -1 mm from the liquid.

The liquid type is Organic and the expected labware type is Plate_96_F_Vis_Greiner.

---------------------------------------------------------------------------

Span-8 Wash

Wash all probes of Pod2 at AW1; do a passive wash by dispensing 4 mL while in the wells and 2 mL to waste. Delay for 300 ms after each dispense.

---------------------------------------------------------------------------

- **End Loop**

---------------------------------------------------------------------------

**End Group**

---------------------------------------------------------------------------

**Move Labware**

Move the entire stack of labware at "P6" to "P5" using pod "Pod1".

---------------------------------------------------------------------------

**Group: Lecture**

---------------------------------------------------------------------------

Move Labware

Move the entire stack of labware at "P9" to "inner1" using pod "Pod1".

---------------------------------------------------------------------------

Device: CustomConveyor

Move toward deck? False

Read bar code?: False

Retries: 2

Stop Run on error?: Abort

Read bar code for decision making?: False

Simulation bar code:

---------------------------------------------------------------------------

BRT Module: BRT

Move From: outer1

Move To: MultimodeReader1

Grip: Narrow

Open Source Position? True

Move to safe position before opening Source position? True

Open Destination Position? True

Close Source Position? True

Close Destination Position? True

---------------------------------------------------------------------------

**SpectraMaxI3: Run a predefined protocol**

---------------------------------------------------------------------------

BRT Module: BRT

Move From: MultimodeReader1

Move To: outer1

Grip: Narrow

Open Source Position? True

Move to safe position before opening Source position? True

Open Destination Position? True

Close Source Position? True

Close Destination Position? True

---------------------------------------------------------------------------

Device: CustomConveyor

Move toward deck? True

Read bar code?: False

Retries: 2

Stop Run on error?: Abort

Read bar code for decision making?: False

Simulation bar code:

---------------------------------------------------------------------------

Move Labware

Move the entire stack of labware at "inner1" to "P9" using pod "Pod1".

---------------------------------------------------------------------------

Move Labware

Move the entire stack of labware at "P10" to "P9" using pod "Pod1".

---------------------------------------------------------------------------

**End Group**

---------------------------------------------------------------------------

**Span-8 Wash**

Wash all probes of Pod2 at AW1; do a passive wash by dispensing 8 mL while in the wells

and 2 mL to waste. Delay for 300 ms after each dispense.

---------------------------------------------------------------------------

**Finish**

Method completed.

Remove the tips from all pods. Clear all labware from the deck. Clear all labware from SILAS devices. Clear all global variables.

---------------------------------------------------------------------------

- **Spectrophotometric measurement**

For spectrophotometric measurement, the SpectraMax i3 was set in Kinetic mode, with 5 measurement over 6 minutes (1.5 min per point). For each measurement, three wavelengths were used, 405 nm, 440 nm and 590 nm, corresponding to the pNP-substrates, the AZO-casein, and the other AZO-substrates respectively. The plate type was set to a Greiner SBS 96-Flatwells polystyrene plate. A 60 seconds orbital shaking at medium intensity was performed before measurement to homogenate the wells. No shaking was performed between the readings. Readings were performed in rows. In the end, the average OD value was calculated to smooth the possible OD variation occurring over the 6 minutes.


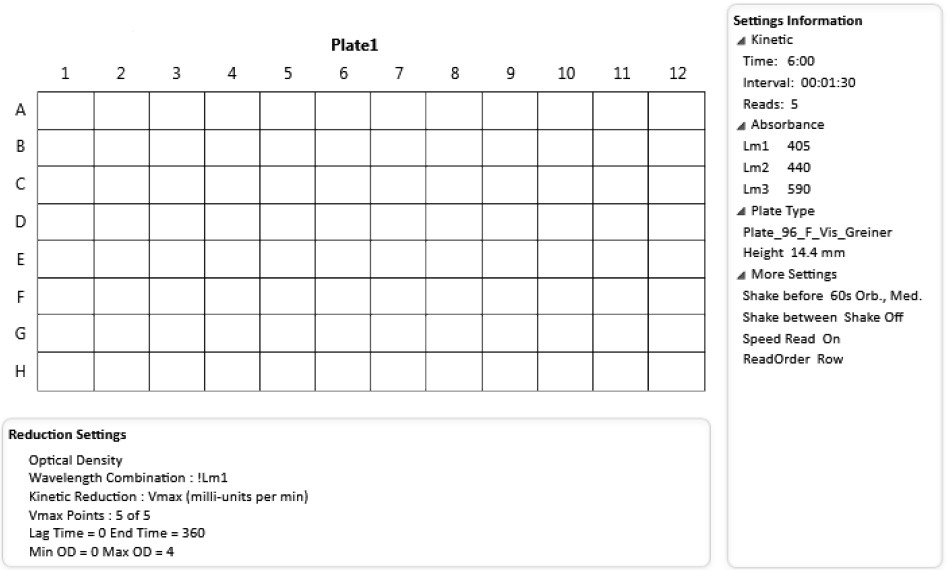


Fig. 6: Parameters of the SpectraMax i3 for spectrophotometric measurement
